# Refraining from Packed Red Blood Cells in Cardiopulmonary Bypass Priming as a Method of Neuroprotection in Pediatric Cardiac Surgery

**DOI:** 10.3390/jcm12041465

**Published:** 2023-02-12

**Authors:** Artem A. Ivkin, Evgeny Grigoriev, Anna V. Sinitskaya

**Affiliations:** Research Institute for Complex Issues of Cardiovascular Diseases, 650002 Kemerovo, Russia

**Keywords:** children, neuroprotection, systemic inflammatory response, cardiac surgery

## Abstract

Congenital heart defect (CHD) surgeries are performed with cardiopulmonary bypass (CPB) and are complicated by several factors that affect the child’s brain. However, to date, the number of studies on brain protection in cardiac surgery remains small. The aim of this study was to assess the impact of refraining from using packed red blood cells (PRBCs) in priming solutions in children with congenital defects (CHDs) who require surgical interventions using CPB to prevent brain injury in the postoperative period. Material and methods: This study included 40 children, and the mean age was 14 (12–22.5) months and the mean weight was 8.8 (7.25–11) kg. All patients underwent CHD closure using CPB. The patients were divided into two groups depending on the use of PRBCs in the priming solution. Brain injury was assessed using three specific blood serum markers, namely S100 calcium-binding protein β (S100β), neuron-specific enolase (NSE) and glial fibrillary acidic protein (GFAP) before surgery, after the completion of CPB and 16 h after surgery (first, second and third control points). Markers of systemic inflammatory response were also analyzed, including interleukin-1, -6, -10 and tumor necrosis factor alpha (TNF-α). A clinical assessment of brain injury was carried out using a valid, rapid, observational tool for screening delirium in children of this age group, i.e., “Cornell Assessment of Pediatric Delirium”. Results: Factors of the intra- and postoperative period were analyzed, such as hemoglobin levels, oxygen delivery (cerebral tissue oxygenation, blood lactate level and venous oxygen saturation) and indicators of organ dysfunction (creatinine, urea, bilirubin levels, duration of CPB and length of stay in the ICU). Following the procedure, there were no significant differences between the groups and all indicators were within the reference values, thus demonstrating the safety of CHD closure without transfusion. Moreover, the highest level of specific markers of brain injury were noted immediately after the completion of CPB in both groups. The concentration of all three markers was significantly higher in the group with transfusion after the completion of CPB. Moreover, GFAP levels were higher in the transfusion group and 16 h after surgery. Conclusions: The results of the study show the safety and effectiveness of brain injury prevention strategies that consist of not conducting PRBC transfusion.

## 1. Introduction

The number of conducted closures of congenital heart defects (CHDs) in children increases worldwide every year. Most of them are performed using cardiopulmonary bypass (CPB) and all of them pathologically affect various organs and systems, especially the brain. Brain structure at the cellular level is represented by a neurovascular unit (NVU), and systemic inflammatory response syndrome (SIRS) induced during surgery manifests as neuroinflammation [1,2]. At the same time, the factors that intraoperatively initiate SIRS are diverse and include hemodynamic instability, ionic and acid-base disorders, abnormal arterial blood gas, duration and difficulty of surgery, the effect of anesthesia and the use of sympathomimetic drugs [3,4,5]. Such factors can occur during any kind of surgery, and they are typical for cardiac surgery; however, cardiovascular procedures also require CPB, which involves an even higher number of pathological factors. Among them, it is possible to distinguish direct brain injuries, for example, microembolism, hypoxia and laminar flow [6,7,8]. The second group consists of factors capable of initiating SIRS, including contact of blood with nonbiological surfaces during CPB, blood–air interface in the CPB circuit, hemolysis, hemodilution, hypothermia or hyperthermia. Realized as neuroinflammation within the brain, SIRS disrupts and damages NVU [4,9,10,11]. This is due to the blood–brain barrier, which normally protects brain neurons from systemic inflammation. When this barrier is affected by any factors that can directly damage the brain, a phenomenon, such as the effect of systemic inflammatory cytokines, may occur. In addition, the activation of microglia and astrocytes, as well as the direct production of inflammatory mediators in the brain tissue, also increase further damage [12].

At the same time, it should be noted that SIRS initiated during surgery can persist for 3 days after and can damage brain autoregulation. As a result, this damage to autoregulation leads to a decrease in cerebral perfusion with subsequent cerebral hypoxia and associated cognitive dysfunctions [13].

Children’s age and body weight up to 10 kg are proven risk factors for the development of SIRS during cardiac surgery [14]. In children, especially newborns, the critical level of hemodilution is inevitable due to the discrepancy between the CPB circuit volume and the child’s blood volume, which is another risk factor because it can lead to hypoxia. A blood transfusion is performed in order to prevent it; however, transfusion, as well as hypoxia, can negatively affect the brain. Recent studies showed that every 10 mL/kg of intraoperative transfusion of packed red blood cells (PRBCs) increases the likelihood of postoperative delirium (POD) by 90% [15]. The pathophysiological mechanism of this phenomenon lies in the fact that the donated blood, being foreign to the recipient, initiates and enhances SIRS, which is realized via neuroinflammation and NVU damage [16,17]. In addition, the use of donor blood components is dangerous due to infections that can be transmitted to the recipient during transfusion [18]. The risk of transfusion complications, such as transfusion-related lung injury, transfusion-related immunomodulation and transfusion-associated circulatory overload [19], also have a negative effect.

Thus, both the transfusion itself and the risk of hypoxia when transfusion is limited are potentially equally dangerous for the patient, which makes this problem relevant for pediatric anesthesiology. Unfortunately, the number of works on this topic is limited. That is why the aim of our study was to prove that the strategy of minimizing the use of donated red blood cells is safe. In addition, taking into account the fact that transfusion itself is a risk factor for SIRS, the aim of the study was also to find out whether abstaining from it is an effective method of cerebroprotection.

In order to confirm brain damage, there is a method used to evaluate the concentration of specific markers in blood plasma, which are the most studied in the pediatric population, namely the S100β protein, neuron-specific enolase (NSE) and glial fibrillar acid protein (GFAP). The S100β protein is physiologically located in glial astrocytes and is detected in the blood only when the integrity of the BBB is compromised; its concentration correlates with the volume of affected brain tissue [20,21]. NSE is of interest primarily because it is an intracellular tissue-specific enzyme of neurons and its presence in the blood serum points to a violation of integrity [22,23]. Elevated GFAP levels in blood and cerebrospinal fluid indicate a violation of the integrity of astrocyte membranes and suggest BBB dysfunction [24].

In order to clarify the severity of SIRS, one can use the assessment of the level of specific markers in blood plasma, such as interleukin 1 (IL-1), interleukin 6 (IL-6), interleukin 10 (IL-10) and tumor necrosis factor alpha (TNF-α). According to numerous studies, the blood serum levels of the above-mentioned markers make it possible to assess the degree of SIRS severity [25,26].

## 2. Materials and Methods

The study was conducted at the Department of Anesthesiology and Resuscitation, Research Institute for Complex Issues of Cardiovascular Diseases. The study included 40 children aged 6 to 36 months. The mean age was 14 (12–22.5) months, and the mean weight was 8.8 (7.25–11) kg. All children underwent closure of ventricular and atrial defects with CPB. The power analyses were carried out according to the following formula:

n = (𝑡^2^ × P × Q)/∆^2^,

where 𝑡 is the critical value of Student’s distribution at the appropriate level of significance (in this study, it was 0.05); ∆ is the maximum permissible error (%); P is the rate of cases in which the studied trait occurs (%); Q is the rate of cases in which the analyzed trait does not occur (100-𝑃). According to the calculation, the study was supposed to include 196 patients, but this was only a pilot study. The prospective randomized study protocol was approved by the Institutional Review Board of the Research Institute (protocol no. 20 dated 20 November 2018).

Upon inclusion to the study, patients underwent randomization using the sealed envelope method as followed:

Treatment group: crystalloid and colloid-based priming without PRBCs (w/oRBC, 20 patients);

Control group: crystalloid and colloid-based priming with PRBCs (wRBC, 20 patients). 

The characteristics of the patients by groups are given in Table 1.

The following Cloud-Clone Corp. (Export Processing Zone, Wuhan, Hubei, China) markers were used to diagnose NBE damage: S100β (SEA567Hu); NSE (SEA537Hu); and GFAP (SEA068Hu). The following markers were used to diagnose SIRS: IL-1 (HEA563Hu); IL-6 (HEA079Hu); IL-10 (SEA056Hu); and TNF-α (HEA133Hu). For analyses, the ELISA method using the «Titramax-1000» (Heidolph Instruments GmbH & Co. Schwabach, Germany) device was chosen. 

Blood analyses were carried out in the following three stages: stage 1 occurs upon transfer to the operating room, after catheterization and before induction of anesthesia; stage 2 occurs immediately after the completion of CPB; and stage 3 occurs 16–18 h after surgery. Blood samples were collected from the internal jugular vein. 

The clinical marker of NVU damage in our study was defined as the presence and severity of postoperative delirium (POD), assessed using the Cornell Assessment for Pediatric Delirium (CAPD), validated for pediatric cardiac surgery patients [27]. A score of 9 or above indicates the presence of delirium. Assessment was carried out on the first day after surgery in the Department of Anesthesiology and Resuscitation, only after extubation and with independent breathing through the natural respiratory tract. In order to exclude errors in assessment due to agitation, this was carried out 2 h after extubation. The children were assessed using the RASS scale, and if the score was −4 or −5, further assessment was not carried out until the level of consciousness returned to −3 or more, thus excluding the influence of depression of consciousness [28]. Moreover, a preliminary evaluation using pain assessment scales to exclude the influence of pain on the result of the CAPD was performed. The following scales were used: the Neonatal Infant Pain Scale (NIPS) for children under 1 year old [29]; and the Face, Legs, Activity, Cry, and Consolability (FLACC) scale for children 1 to 3 years old [27]. A score of more than 3 on the scales indicated pain. If pain was detected during evaluation, then analgesia was performed with subsequent re-evaluation. Assessment of POD was carried out only when no pain was detected. 

Given that there was a risk of hypoxia in the w/oRBC group due to lower hemoglobin levels, the safety of the patient was of extreme importance. In order to ensure safety, a number of laboratory and instrumental indicators were monitored at all stages of surgery and 16–18 h after it. The levels of hemoglobin, hematocrit and RBCs were analyzed. The assessment of oxygen delivery and consumption by tissues was carried out using venous oxygen saturation, blood lactate level and cerebral oximetry indicators; moreover, pulse oximetry data were also evaluated. The level of urea and creatinine was recorded on the first postoperative day in order to monitor renal function, and direct and indirect bilirubin was recorded to monitor liver health. Moreover, the level of white blood cells was recorded. The assessment of the immediate postoperative period, in addition to all of the above, also included blood loss in drainage, the duration of lung ventilation and the length of stay in the ICU. The frequency of use and dosing of inotropic drugs at all stages of the study was also analyzed.

### Statistical Analyses

Statistical data processing was carried out using BioStat Pro 5.9.8 software. To address non-normality to data (Shapiro–Wilk test, *p* < 0.05), nonparametric analysis methods were used. The data are presented as median (Me) and upper (Q1) and lower quartiles (Q3). The Mann–Whitney test was used for the comparative analysis of the quantitative variables [30]. A comparative analysis of the qualitative variables was carried out using a 2 × 2 conjugation table. The differences were considered statistically significant at *p* < 0.05.

## 3. Results

### 3.1. Evaluation of the Hemoglobin Level and Its Oxygen Saturation in the Intraoperative Period

Characteristics of the intraoperative period are given in Table 2. The groups differed in hemoglobin and hematocrit levels; higher indicators were present in the control group. Indicators of venous oxygen saturation did not differ during CPB; however, by the end of surgery, it was lower in the w/oRBC group, i.e., 71% (69.8–73) vs. 73% (71.8–77) (*p* = 0.01). There were no differences in blood lactate level between the groups. The indicators of pulse oximetry at all stages of surgery also did not differ significantly. Cerebral oxygenation differed only at the end of surgery and higher values were noted in the controls, as follows: 70.5% (69.8–75) vs. 77% (74.5–78) (*p* = 0.008). Hemodynamic parameters were similar in both groups, as demonstrated by the absence of episodes of hypotension and the difference in inotropic support.

### 3.2. Evaluation of the Levels of Hemoglobin and Hematocrit, Venous Blood Saturation, Blood Lactate Level, WBC, Blood Creatinine and Urea Level in the Postoperative Period

Characteristics of the postoperative period are given in Table 3. In the postoperative period, the level of hemoglobin and hematocrit was significantly lower in the w/oRBC group, as was the level of RBC. The saturation of venous blood was 70% (68.8–73.3) vs. 76.5% (73–80) (*p* < 0.001), and higher values were noted in the wRBC group. Blood lactate levels remained the same in the intraoperative period and did not differ between groups. There was a statistically significantly higher level of WBCs in the wRBC group as follows: 8.5 ∗ 10^9^ (7.9–11.1) vs. 10.8 ∗ 10^9^ (9.3–12.8) (*p* = 0.013). It is worth noting that the level of direct bilirubin between the groups did not differ, while the level of indirect bilirubin in the blood was significantly higher in the controls (3.8 mmol/L (2.7–4.9) vs. 9.5 mmol/L (4.9–13) (0.001)). Blood creatinine levels in the postoperative period were 26.5 mmol/L (19.8–31) in the w/oRBC group and 32.5 mmol/L (26–40) in the wRBC group (*p* = 0.015). The blood urea levels were as follows: 3.7 mmol/L (3.1–4.9) in the w/oRBC group and 4.5 mmol/L (4–5.5) in the wRBC group (*p* = 0.032). Thus, both indicators of renal function prevailed in the wRBC group in the intraoperative period. There were no differences between the groups in the duration of inotropic support and lung ventilation, the length of stay in the ICU and blood loss in drainage.

### 3.3. Evaluation of Brain Injury Marker Levels

Dynamics of markers are given in Table 4. The results of the analysis of S100β concentration at various stages of surgery show that the highest concentration (w/oRBC: 522.1 ng/mL; wRBC: 947.7 ng/mL) was observed after the completion of CPB. However, 16 h after surgery, the concentration was significantly lower (w/oRBC: 167 ng/mL; wRBC: 207.7 ng/mL) than before surgery (w/oRBC: 185.3 ng/mL; wRBC: 244.2 ng/mL). Analyses of the intergroup difference revealed differences only after the completion of CPB.

The highest concentration of NSE in both groups was noted immediately after the completion of CPB (w/oRBC: 30.51 ng/mL; wRBC–44.92 ng/mL), which decreased 16 h after surgery (w/oRBC: 19.85 ng/mL; wRBC: 24.15 ng/mL). It is worth noting that it remained significantly higher compared to baseline (w/oRBC: 19.85 ng/mL; wRBC: 24.15 ng/mL). Statistical analyses showed that a higher concentration of the marker was noted in the wRBC group.

GFAP concentration dynamics are similar to the above-mentioned markers. It reached its highest point after the completion of CPB in both groups (w/oRBC: 0.1172 ng/mL; wRBC: 0.1288 ng/mL), but it remained elevated compared to baseline 16 h after intervention only in the wRBC group (w/oRBC: 0.11 ng/mL; wRBC: 0.11212 ng/mL). Statistically significant differences between the groups were characteristic for the II and III stages of the study.

### 3.4. Evaluation of the SIRS Markers Level

Dynamics of markers are given in Table 4. The highest IL-1 concentration occurred at the second control point, i.e., after the completion of CPB (w/oRBC: 2.86 ng/mL; wRBC: 3.3 ng/mL) in both groups, it was significantly higher (*p* < 0.001) compared to baseline (w/oRBC: 2.57 ng/mL; wRBC: 2.58 ng/mL). A total of 16 h after surgery, the concentration of the marker decreased, but it still remained significantly higher compared to baseline (*p* < 0.001) (w/oRBC: 2.72 ng/mL; wRBC: 2.82 ng/mL). The intergroup comparison revealed a statistically significant difference only at the second control point (*p* = 0.003) with a higher IL-1 concentration in the wRBC group.

Unlike the previous marker, the peak concentration of IL-6 was noted at the third control point in both groups. The dynamics of IL-6 concentration were as follows: it was significantly higher in both groups compared to baseline (w/oRBC: 2.47 ng/mL; wRBC: 2.64 ng/mL) after the completion of CPB (w/oRBC: 29.1 ng/mL; wRBC: 27.58 ng/mL), and on the morning after surgery (w/oRBC: 31.56 ng/mL; wRBC: 48.91 ng/mL) (*p* < 0.001). There were no intergroup differences; however, a tendency toward statistically significant differences was noted at the third control point (*p* = 0.087).

IL-10 concentration was significantly higher compared to baseline (w/oRBC: 0.62 ng/mL; wRBC: 0.62 ng/mL) upon the completion of CPB (w/oRBC: 7.92 ng/mL; wRBC: 8.78 ng/mL) (*p* < 0.001). However, on the next day in the w/oRBC group, its level (0.69 ng/mL) did not differ from baseline (*p* = 0.49), unlike in the wRBC group (0.8 ng/mL), where it was significantly higher (*p* = 0.006). The intergroup difference was revealed 16 h after surgery—the level of IL-10 was significantly higher in the wRBC group (*p* = 0.005). There was a tendency (*p* = 0.07) toward higher (but not significant) levels of the marker in the wRBC group after the completion of CPB.

The TNF-α marker did not differ between the groups of patients at baseline (w/oRBC: 1.29 ng/mL; wRBC: 1.21 ng/mL) (*p* = 0.19), and the highest concentration was observed in both groups at the second control point (w/oRBC: 1.33 ng/mL; wRBC: 1.81 ng/mL); however, in the w/oRBC group, it did not differ significantly from baseline (*p* = 0.21) compared to the wRBC group (*p* = 0.006). At the third control point, the TNF-α values in the w/oRBC group did not differ from baseline, but in the wRBC group, TNF-α was higher after the completion of CPB (*p* = 0.034).

### 3.5. Diagnosis of Delirium

**Figure 1:**
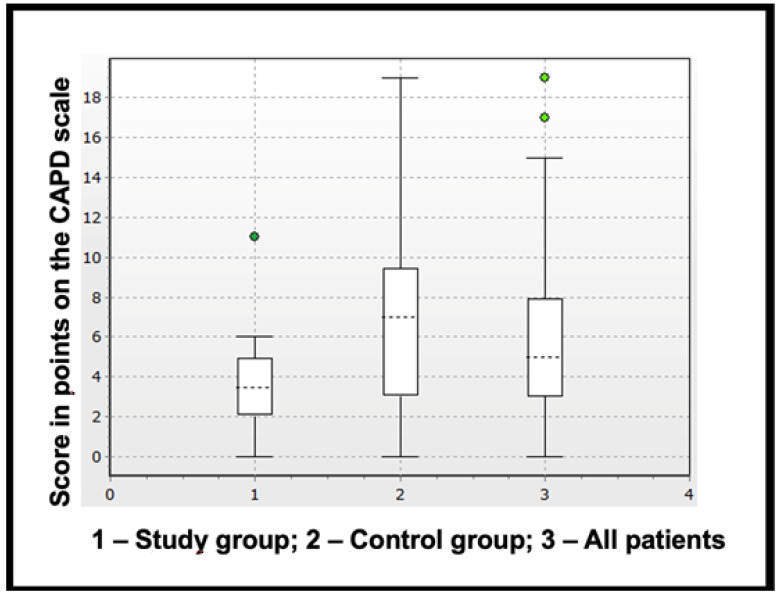
Score in points on the CAPD scale.

POD in was detected in nine children (22.5%), and the average score on the CAPD scale was 5 (3–8). In the w/oRBC group, POD was diagnosed in two children (10%) with a score of 3.5 (2.5–5). In the wRBC group, it was detected in seven patients (35%) and the score was 7 (3–9.25), showing a statistically significant intergroup difference in the average score (*p* = 0.026), (Figure 1).

In all patients who were diagnosed with delirium, it regressed no later than on the third day after the operation. All patients included in the study were transferred from the intensive care unit to the cardiac surgery unit and discharged from the hospital in a satisfactory condition. They are currently under the supervision of a pediatric cardiologist. In further studies, we plan to show the results of assessing the cognitive status of this group of patients one year after surgery.

## 4. Discussion

Patients differed significantly in hemoglobin and hematocrit levels, which can be explained by the priming of CPB with PRBC only in the controls. Despite the lower oxygen capacity in the w/oRBC group, the level of oxygen transport remained sufficient at all stages of the procedure, as evidenced by the reference values of venous oxygen saturation during the entire follow-up period, as well as the absence of differences between the groups in blood lactate concentration. It must be mentioned that brain oxygenation was significantly higher in the controls, which is explained by the higher average hemoglobin level in the wRBC group in the blood transfusion patients. However, the absolute values of NIRS (Near-infrared spectroscopy) might be not that important. According to the literature, a key role in predicting brain injury is played by a decrease in baseline (preoperative) indicators. Thus, a 20% decrease [31] or, according to some data, even a 10% decrease in NIRS compared to baseline is dangerous [32]. In all our patients, only an increase in cerebral oxygenation was recorded at all stages of surgery, which indicated a sufficient level of oxygen supply to the brain. Taking into account the fact that all oxygen transport parameters remained within the reference values in the postoperative period, we can state that the strategy of “bloodless” perfusion is safe in terms of the balance of oxygen delivery and consumption. It can be proven by the absence of organ dysfunctions in both groups. The level of bilirubin was within the reference values and liver function was stable, but it is worth noting that the wRBC group had higher levels of bilirubin. Perhaps this was the reason for bilirubin increase, and it may indicate an active process of hemolysis in donor RBCs [33]. Renal function was also stable.

Considering the problem of neuroinflammation, higher levels of blood leukocytes in the controls is a point of interest. The number of WBCs serves as an indicator of the severity of SIRS and, because statistically higher concentrations of SIRS markers were noted in the wRBC group, we can point to the faster development of SIRS in the controls, which underlies neuroinflammation and brain injury [34,35]. It is possible to confirm this hypothesis with the help of specific markers of brain injury. The highest concentration of S100β was noted after the completion of CPB. Because its half-life is about 1 h [36], it can be assumed that the increase in its concentration is due to CPB [37,38]. Taking into account that there were no differences between the groups in the pre- and intraoperative periods, it can be concluded that it was the transfusion of blood that caused an increase in the level of S100β in the controls by initiating and potentiating SIRS that was followed by a neuroinflammatory reaction.

This theory is also supported by the fact that the highest concentration of NSE in both groups was noted immediately after the completion of CPB, which is explained by the influence of its factors on the direct and indirect damage to neurons via SIRS. After 16 h, the concentration of the marker decreases, but it remains elevated compared to baseline. This may indicate a continuing brain injury. Moreover, NSE and the above-mentioned marker were statistically higher in patients with transfusion. GFAP concentration in the blood was similar to S100β and NSE and was an additional confirmation of the theory ascribing transfusion to the pathological factors affecting the brain. The only difference is that, unlike other markers, its concentration is significantly higher after surgery, which may indirectly indicate ongoing brain injury in the postoperative period in patients with intraoperative transfusion.

Overall, POD was detected in 22.5% of patients. Given that the number of similar studies is small, it is difficult to make a proper comparison. Moreover, analyses of their structure makes it even harder. For example, one of the largest studies is the work by Anita K. Patel [39], which included patients from birth to 21 years of age with various heart surgeries for CHD. The incidence of POD was 49%. Despite the long follow-up, there was a significant disadvantage—children with an RACHS score (risk adjustment for congenital heart surgery) of 1 to 6 were included. In other words, the assessment of POD in children was conducted without taking into account the individual characteristics of hemodynamics, which ultimately affected the result [40], but this in no way detracted from the significance of the study. Similar data were obtained independently in another study of these group of patients, where POD was detected in 57% [41]. Thus, this issue remains a relevant topic to study as the scientific literature still does not have substantial data on the assessment of children by CHD type and their correction with ranking. Regarding the level and severity of POD, a significant difference in scores between the groups on the CAPD scale seems quite natural given the higher level of brain injury in the controls according to the analyses of markers.

Work can be limited by a small number of control points in the analyses of the concentration of the studied markers in the blood. According to their dynamics, all of them have a different concentration peak from the moment of initiation of their release (CPB). In particular, NSE has a high diagnostic value of 24, even 36 h after the moment of cerebral injury [42]. Regarding GFAP, we also want to note that there are studies demonstrating its high concentrations after 24 [43] and even 72 h [44] from the onset of the brain damage factor. We did not observe any organ damage among the patients; therefore, we decided to monitor organ dysfunction for more than 24 h after surgery.

Our results are consistent with many other studies in which the negative effects of transfusion have been proven [45]. However, it should be taken into account that in our research, only the strategy of managing patients with septal congenital heart disease was studied. For other, more complex CHDs, more research may be needed in the future. A good prospect is the development of new techniques that would allow CPB to be performed without using donor blood components in procedures for any, even complex, defects and in children with low body weight.

## 5. Conclusions

The strategy based on not using PRBC in the priming of CPB for the surgical correction of congenital heart disease in children in the intra- and postoperative period seems to be safe. Moreover, this strategy could be effective in reducing SIRS and brain injury. Children undergoing a surgical correction of congenital heart diseases without PRBC seem to have more likely lower levels of postoperative delirium and lower scores on the CAPD scale.

## Figures and Tables

**Table 1:** Characteristics of the studied patient groups.

Investigated Characteristic		Study Group	Control Group	*p*
Number of patients		20 (50%)	20 (50%)	1
Male		7 (35%)	9 (45%)	0.52
Female		13 (65%)	11 (55%)	0.52
Age (months)		15 (12–23.3)	13 (11–21.3)	0.27
Height (cm)		81 (76–86)	75 (71.3–84.3)	0.14
Body weight (kg)		10.5 (9.2–11.3)	9.2 (8.7–11.8)	0.15
Laboratory indicators of blood before surgery				
Leukocyte level (×10^9^/L)		7.4 (6.6–7.9)	7.5 (7–9)	0.17
Erythrocyte level (×10^12^/L)		4.6 (4.5–4.75)	4.6 (3.9–5)	0.7
Hemoglobin level (g/L)		118.5 (115–121.3)	117 (112.8–119)	0.29
Hematocrit level (%)		36 (34–38)	35 (33–37)	0.34
Direct bilirubin level (μmol/L)		2.4 (2.1–3.3)	2.9 (2.1–3.7)	0.54
Indirect bilirubin level (μmol/L)		4.3 (2.5–5.5)	4.5 (2.4–6.7)	0.68
Creatinine level (μmol/L)		38.5 (30.5–44.3)	31 (24.3–43.3)	0.23
Urea level (mmol/L)		3.8 (3.4–4.3)	4 (3–5)	0.98
Preoperative NGAL concentration (ng/mL)		49.19 (24.3–100.1)	45.98 (34.58–98.98)	0.3
Surgical intervention				
Diagnosis	ASD	15 (75%)	15 (75%)	1
	VSD	5 (25%)	5 (25%)	1
Surgical approach	Median sternotomy	14 (70%)	15 (75%)	0.85
	Side sternotomy	6 (30%)	5 (25%)	0.85
Duration of surgery		196 (188–203)	189 (181–200)	0.3
CPB duration (min.)		40.5 (33–47)	45 (35–49.5)	0.5
Duration of aortic clamping (min.)		27.5 (20.3–33)	29 (22.3–36.3)	0.59

**Table 2:** Characteristics of the factors of the intraoperative period.

Investigated Characteristic	Study Group	Control Group	*p*
Laboratory indicators			
Hemoglobin level during the CPB (g/L)	87 (81.0–91.3)	92 (87.3–97.3)	0.008
Hematocrit level during the CPB (%)	25.5 (24.0–27.0)	29 (27.8–31.0)	˂0.001
Hemoglobin level at the end of the operation (g/L)	106.0 (101.8–110.3)	130.5 (104.0–125.5)	˂0.001
Hematocrit level at the end of the operation (%)	31.5 (30–33.3)	40.0 (38.8–41.5)	˂0.001
Venous blood saturation during the CPB (%)	85.0 (83.8–89.0)	88.5 (86.0–90.0)	0.26
Venous blood saturation at the end of the operation (%)	71.0 (69.8–73.0)	73.0 (71.8–77.0)	0.01
Blood lactate during the CPB (mmol/L)	1.5 (1.3–1.8)	1.5 (1.2–1.9)	0.87
Blood lactate at the end of the operation (mmol/L)	1.5 (1.3–1.7)	1.5 (1.2–1.7)	0.46
Preoperative NGAL concentration (ng/mL)	49.2 (24.3–100.1)	46.0 (34.6–99.0)	0.3
Monitoring indicators			
SpO_2_ indicators before the operation (%)	97.0 (90.5–98.0)	98.0 (95.5–98.5)	0.33
SpO_2_ indicators at the end of the operation (%)	99.0 (98.0–99.0)	99.0 (99.0–100.0)	0.03
rSO_2_ indicators before the operation (%)	65.0 (61.5–73.5)	67.0 (61.5–70.5)	0.77
rSO_2_ indicators during the CPB (%)	83.0 (80.5–86.5)	85.0 (81.5–87.0)	0.40
rSO_2_ indicators at the end of the operation (%)	70.5 (69.8–75.0)	77.0 (74.5–78.0)	0.008
Inotropic drugs			
Number of patients with nootropic drugs	4 (20%)	5 (25%)	0.7
Hydrobalance indicators			
Intravenous infusion volume (ml/kg)	15.6 (13.5–16.4)	15.7 (12.8–17.4)	0.31
Diuresis volume (ml/kg)	11.0 (9.0–12.4)	10.5 (9.3–12.3)	0.43
Ultrafiltration volume during CPB (ml/kg)	11.0 (10.1–13.3)	11.7 (10.2–13.5)	0.37

**Table 3:** Characteristics of the factors of the postoperative period.

Investigated Characteristic	Study Group	Control Group	*p*
Laboratory indicators			
Hemoglobin level (Γ/л)	101.0 (98.8–107.0)	124.0 (113.0–127.0)	˂0.001
Hematocrit level (%)	30.0 (29.0–32.0)	34.0 (33.0–36.0)	˂0.001
Venous blood saturation (%)	70.0 (68.8–73.3)	76.5 (73.0–80.0)	˂0.001
Blood lactate (mmol/L)	1.2 (1.1–1.35)	1.2 (1.08–1.3)	0.67
Red blood cell level (×10^12^)	3.8 (3.6–4.1)	4.8 (4.5–5.0)	˂0.001
Leukocyte level (×10^9^)	8.5 (7.9–11.1)	10.8 (9.3–12.8)	0.013
Direct bilirubin level (μmol/L)	2.9 (2.2–3.2)	3.3 (2.3–4.4)	0.29
Indirect bilirubin level (μmol/L)	3.8 (2.7–4.9)	9.5 (4.9–13.0)	˂0.001
Creatinine level (μmol/L)	26.5 (19.8–31.0)	32.5 (26.0–40.0)	0.015
Urea level (mmol/L)	3.7 (3.1–4.9)	4.5 (4.0–5.5)	0.032
Postoperative NGAL concentration (ng/mL)	87.3 (41.3–159.1)	74.5 (49.5–136.2)	0.46
Dynamic observation indicators			
Drainage losses in the first day after surgery (ml/kg)	54.6 (46.4–84.0)	68.0 (53.3–82.4)	0.3
Duration of stay in the intensive care unit (hours)	23.5 (21.0–29.0)	23.0 (21.8–41.5)	0.97
Duration of mechanical ventilation (hours)	7.0 (6.0–8.0)	8.0 (6.8–9.0)	0.34
Inotropic drugs			
Number of patients with nootropic drugs	4 (20%)	5 (25 %)	0.7
Hydrobalance indicators			
The volume of fluid injected during the period of stay in the intensive care unit (ml)	64.0 (62.70–69.2)	61.0 (59.4–64.9)	0.1
The volume of diuresis during the period of being in the intensive care unit (ml)	24.0 (22.0–26.5)	28.0 (22.5–30.0)	0.08

**Table 4:** Dynamics of markers.

Investigated Characteristic	Study Group	Control Group	*p*
IL-1b BO, pg/mL	2.6 (2.2–2.8)	2.6 (2.5–3.0)	0.16
IL-1b EO, pg/mL	2.9 (2.7–3.1)	3.3 (3.2–3.5)	0.003
IL-1b 16 h after surgery, pg/mL	2.7 (2.6–3.1)	2.8 (2.7–3.1)	0.46
IL-6 BO, pg/mL	2.5 (2.4–2.7)	2.6 (2.4–5.9)	0.21
IL-6 EO, pg/mL	29.1 (15.5–40.6)	27.6 (16.9–48.5)	0.18
IL-6 16 h after surgery, pg/mL	31.6 (26.8–48.9)	48.9 (33.9–57.6)	0.087
IL-10 BO, pg/mL	0.6 (0.6–0.7)	0.6 (0.6–0.9)	0.39
IL-10 EO, pg/mL	7.9 (4.5–12.1)	8.8 (5.6–38.5)	0.07
IL-10 16 h after surgery, pg/mL	0.7 (0.6–0.8)	0.8 (0.8–1.4)	0.005
TNF-α BO, pg/mL	1.3 (1.1–1.5)	1.2 (1.2–1.3)	0.19
TNF-α EO, pg/mL	1.3 (1.3–1.8)	1.81 (1.4–3.3)	0.034
TNF-α 16 h after surgery, pg/mL	1.2 (1.1–1.6)	1.3 (1.2–1.9)	0.1
S-100-ß BO, ng/m	185.3 (147.05–230.1)	244.2 (165.93–360.18)	0.33
S-100-ß EO, ng/mL	522.1 (386.65–702.9)	947.7 (696.93–1378.25)	*p* ˂ 0.001
S-100-ß 16 h after surgery, ng/mL	167 (95.7–204.8)	207.7 (125.23–291.25)	0.18
NSE BO, ng/m	16.57 (13.39–19.58)	14.51 (12.34–18.47)	0.358
NSE EO, ng/mL	30.51 (22.8–36.99)	44.92 (34.1–55.06])	0.007
NSE 16 h after surgery, ng/mL	19.85 (17.04–24.4)	24.15 (16.67–29.29)	0.494
GFAP BO, ng/m	0.1094 (0.1035–0.1115)	0.1137 (0.1079–0.1242)	0.06
GFAP EO, ng/mL	0.1172 (0.1093–0.1198)	0.1238 (0.1195–0.1348)	0.004
GFAP 16 h after surgery, ng/mL	0.11 (0.105–0.1197)	0.1212 (0.1177–0.1404)	0.002

Note: BO: before operation; EO: end of operation.

## Data Availability

Not applicable.

## Abbreviations

CHD	congenital heart defects
CPB	cardiopulmonary bypass
S100β	S100 calcium-binding protein β
NSE	neuron-specific enolase
GFAP	glial fibrillary acidic protein
PRBCs	packed red blood cells
w/oRBC	without PRBCs
wRBC	with PRBCs
SIRS	systemic inflammatory response syndrome
NVU	neurovascular unit
POD	postoperative delirium
